# Effect of an Injury Awareness Education Program on Risk-Taking Behaviors and Injuries in Juvenile Justice Offenders: A Retrospective Cohort Study

**DOI:** 10.1371/journal.pone.0031776

**Published:** 2012-02-15

**Authors:** Kwok M. Ho, Edward Litton, Elizabeth Geelhoed, Monica Gope, Maxine Burrell, Jacqueline Coribel, Angela McDowall, Sudhakar Rao

**Affiliations:** 1 Department of Intensive Care, Royal Perth Hospital, Perth, Australia; 2 School of Population Health, University of Western Australia, Perth, Australia; 3 School of Medicine and Pharmacology, University of Western Australia, Perth, Australia; 4 Department of Emergency Medicine, Royal Perth Hospital, Perth, Australia; 5 State Trauma Unit, Royal Perth Hospital, Perth, Australia; RAND Corporation, United States of America

## Abstract

**Background:**

Risk-taking behavior is a leading cause of injury and death amongst young people.

**Methodology and Principal Findings:**

This was a retrospective cohort study on the effectiveness of a 1-day youth injury awareness education program (Prevent Alcohol and Risk-related Trauma in Youth, P.A.R.T.Y.) program in reducing risk taking behaviors and injuries of juvenille justice offenders in Western Australia. Of the 3659 juvenile justice offenders convicted by the court magistrates between 2006 and 2010, 225 were referred to the P.A.R.T.Y. education program. In a before and after survey of these 225 participants, a significant proportion of them stated that they were more receptive to modifying their risk-taking behavior (21% before *vs.* 57% after). Using data from the Western Australia Police and Department of Health, the incidence of subsequent offences and injuries of all juvenile justice offenders was assessed. The incidence of subsequent traffic or violence-related offences was significantly lower for those who had attended the program compared to those who did not (3.6% *vs.* 26.8%; absolute risk reduction [ARR] = 23.2%, 95% confidence interval [CI] 19.9%–25.8%; number needed to benefit = 4.3, 95%CI 3.9–5.1; p = 0.001), as were injuries leading to hospitalization (0% *vs.* 1.6% including 0.2% fatality; ARR = 1.6%, 95%CI 1.2%–2.1%) and alcohol or drug-related offences (0% *vs.* 2.4%; ARR 2.4%, 95%CI 1.9%–2.9%). In the multivariate analysis, only P.A.R.T.Y. education program attendance (odds ratio [OR] 0.10, 95%CI 0.05–0.21) and a higher socioeconomic background (OR 0.97 per decile increment in Index of Relative Socioeconomic Advantage and Disadvantage, 95%CI 0.93–0.99) were associated with a lower risk of subsequent traffic or violence-related offences.

**Significance:**

Participation in an injury education program involving real-life trauma scenarios was associated with a reduced subsequent risk of committing violence- or traffic-related offences, injuries, and death for juvenille justice offenders.

## Introduction

Injury is the leading cause of death among young people, responsible for two-thirds of deaths in those under the age of 24 in Australia [1]. Motor vehicle accidents account for the vast majority of these deaths with approximately 14 and 6 motor vehicle accident deaths per 100,000 population-years for males and females, respectively [2].

Injury is also associated with a significant number of hospitalizations, disability and costs. In Western Australia, about 2,500 to 3,000 hospitalizations each year are related to injuries from motor vehicle accidents [3]. The population incidence of head injury in Western Australia is approximately 20 per 100,000 person-years (95% confidence interval: 17–22), resulting in severe disability in a significant number of young people [4], [5]. In 2003, the annual cost of motor vehicle accidents in Australia was approximately $17 billion and accounted for about 2.3% of the Gross Domestic Product [6]. Injury is a global issue. It was estimated that among a total of 288,000 hospitalized survivors of head injuries in the United States in 2003, more than 120,000 patients had long-term disability resulting in substantial demand for rehabilitation services [7].

A significant proportion of assaults and motor vehicle accidents in many developed countries including Australia are related to risky behaviors involving alcohol and drug use. Recreational drugs were detected in the blood of more than 45% of those presenting to a major Australian trauma center following a motor vehicle accident [8]. It has also been estimated that alcohol use is likely to have contributed to between 0.6 and 1.8 serious traffic injuries or fatalities per 1000 population and between 1.4 and 7.7 assaults per 1000 population, every year, in Australia [9].

Health promotion strategies including economic and retailer interventions, alcohol taxation, reducing alcohol availability, legal and legislative strategies, and strategies addressing the servers of alcohol have been shown to be effective in reducing driving under the influence of alcohol [10]. Because each intervention builds on the strengths of every other one, a multi-modality approach has been suggested to be the most effective way to reduce alcohol or drug-impaired driving [10]. Evidence to support the effectiveness of many health education interventions however remains uncertain [10].

The P.A.R.T.Y. (Prevent Alcohol and Risk-related Trauma in Youth) program is a one day youth injury prevention program developed in 1986 by the Regional Trauma Centre at Sunnybrook and Women's College Health Sciences Centre in Toronto, Canada. The program provides relevant information to young people to improve their awareness of injury-producing situations, make informed prevention-oriented choices, and adopt behaviors and actions to minimize risk of injuries.

The P.A.R.T.Y. program has been successfully operating at Royal Perth Hospital, the state of Western Australia's designated major trauma center, since 2006. Traffic-related juvenile justice offenders (aged between 14 and 18 years old) and high school students (between grade 10 and 12) spend a day following the admission of an imaginary major trauma patient. After attending talks on pre-hospital care and the vulnerability of the brain and spinal cord to injuries, participants visit the Emergency Department, Intensive Care Unit (ICU), and Trauma wards. The participants are shown why and when a serious injury is more likely to occur and are given the opportunity of talking to trauma patients about their experiences and attempting to mobilize using a wheelchair and crutches.

In a recent study involving high school students, P.A.R.T.Y program attendance was associated with a reduction in the incidence of alcohol-related driving offences, hospitalizations due to traumatic injuries and deaths [11], [12]. However this study did not assess the effect of the program in changing the attitudes of participants. Furthermore, the generalizability of these results to different regions as well as the efficacy of the P.A.R.T.Y party program in other participant groups remains uncertain.

We hypothesized that this injury education program is effective in reducing risk-taking behaviors and injuries in young people who have committed traffic- or violence-related offences, and conducted a retrospective cohort study to assess the effect of this education program on the attitudes of participants and their risk of subsequent injuries after attending the program.

## Method

This study was approved by the Ethics Committees of Royal Perth Hospital (EC2008/071), Department of Health of Western Australia (2011/2), and Western Australia Police (00000FV001). Informed consent was waived by the ethics committees because of the extreme difficulty in obtaining direct contact with juvenille justice offenders and their families, observational nature of the study and the use of deidentified data. Due to the uncertain effectiveness of this education program, magistrates currently only refer some juvenile justice offenders to the P.A.R.T.Y. education program on an ad hoc basis. When a juvenille justice offender is referred to the P.A.R.T.Y. injury awareness education program, it is considered as one of the many elements of the whole action plan for the offender. There is no set criteria for this referral other than that the overall action plan have to be agreed by the magistrate and juvenille justice offender.

After a short welcome and introduction to familiarize all participants of this education program with the Emergency Response Procedure, a pre-P.A.R.T.Y. program questionnaire was distributed to the participants to ascertain their baseline attitudes and knowledge relating to risk taking behaviors. A second questionnaire on the participants' opinion of injury prevention and evaluation of the P.A.R.T.Y. program was collected at the end of the program to assess whether the participants had changed their perception of risk-taking behaviors.

The study period was March 2006 to December 2010 and included all young people between 14 and 21 years old in Western Australia. Data was obtained from the Western Australia Police for all traffic, alcohol or drug, and violence-related offences. Data obtained from the Department of Health of Western Australia included all hospitalizations related to traffic accidents or violence. The incidence of subsequent offences and injuries in those who had attended the program was compared with those who had not attended the program. The censor date to assess injuries and offences of this study was 1 May 2011.

### Statistical analyses

Categorical and continuous variables with skewed distribution were compared by Chi-square and Mann-Whitney tests, respectively. Multivariate logistic regression analyses were used to identify factors associated with a reduced risk of committing subsequent violence- or traffic-related offences for all juvenile justice offenders. The factors initially analyzed in the multivariate analysis included age, gender, ethnicity, number of prior offences prior to the study period, and IRSD. Variables were removed in a step by step fashion if their associated p-value was >0.25 until only variables associated with a p-value <0.25 were retained in the final model.

We used a propensity score, representing the probability of juvenile justice offenders being selected for the injury education program, to adjust for potential ‘selection’ or ‘referral’ bias. The propensity score for each subject was based on age, gender, ethnicity, number of offences prior to the study period, and socioeconomic background as defined by Index of Relative Socioeconomic Advantage and Disadvantage (IRSD) at a post-code level [13], [14]. A second multivariate model including the propensity score was also used to adjust for the potential effect of selection bias. Finally, the potential interactions between effectiveness of the education program and probability of being referred to the education program was assessed by a calibration plot [15], a technique similar to assessing performance of a prognostic model [16] or interactions between effectiveness of an intervention and severity of illness [17].

Sensitivity analyses were conducted by (i) excluding all individuals who had any committed any offences prior to the commencement of the study, (ii) including only Aboriginal juvenile justice offenders, and (iii) including only subjects from lower socioeconomic background (IRSD<median IRSD of the whole cohort) to tackle the possibility of ‘confounding by indication’ due to selection bias. All statistical tests were two-tailed and performed by SPSS for Windows (version 19, 2010, IL, USA). A p-value<0.05 was taken as statistically significant.

## Results

During the study period, there were 8869 hospitalizations and 113 deaths due to violence- or traffic-related injuries among those aged between 14 and 21 in Western Australia. The mean, median, standard deviation, interquartile range (IQR) and range of their length of hospital stay were 4.6, 1, 12.3, 1–3 and 1–310 days, respectively. A total of 320 patients (3.6%) needed ICU admission with an average length of stay in ICU of 6 days.

Of the 3659 juvenile justice offenders sentenced by court magistrates during the study period, 225 were referred to the P.A.R.T.Y. education program. In the before and after survey of these 225 participants, a significant proportion of them stated that the program would modify their attitude on risk-taking behaviors (21% before *vs.* 57% after, p<0.001). Many written comments from the participants of the program were also very positive about the education program (see Supporting Information file). Court magistrates selected significantly more males, subjects without prior offences, and European or Aboriginal subjects to attend the injury prevention program (**Table 1**). Age, nature of offences and socioeconomic factors appeared to be not different between those who were referred to the education program and those who were not.

**Table 1:** Characteristics of juvenile justice offenders who had or had not been referred to attend the injury prevention program.

Variables	Referred (n = 225)	Not referred (n = 3434)	P value
Age, years (SD, IQR)	16.3 (2.0, 16–17)	16.0 (1.6, 15–17)	0.089
Male, no. (%)	191 (85)	2261 (66)	0.001#
Ethnic groups, no. (%)			0.001#
**-** Caucasian	103 (46)	1082 (32)	
**-** Aboriginal	42 (19)	391 (11)	
**-** Asian	7 (3)	24 (0.7)	
**-** Others	19 (8)	67 (2)	
**-** Unknown	54 (24)	1870 (54)	
Nature of offencesleading to 1^st^ referralto juvenille justicesystem during the studyperiod, no. (%):			0.221
- assault-related	55 (24.4)	973 (28.3)	
- traffic-related	170 (75.6)	2461 (71.7)	
Postcode level socioeconomicindex (IRSD)(SD, IQR)	1009 (53, 973–1049)	1004 (64, 964–1049)	0.142#
Prior offences before the study period, no.(SD, IQR)	0 (0,0–0)	0.1 (0.5, 0–0)	0.001

# p value generated by Chi-square or Mann-Whitney test. SD, standard deviation. IQR, interquartile range. IRSD, Index of Relative Socioeconomic Advantage and Disadvantage.

**Figure 1:**
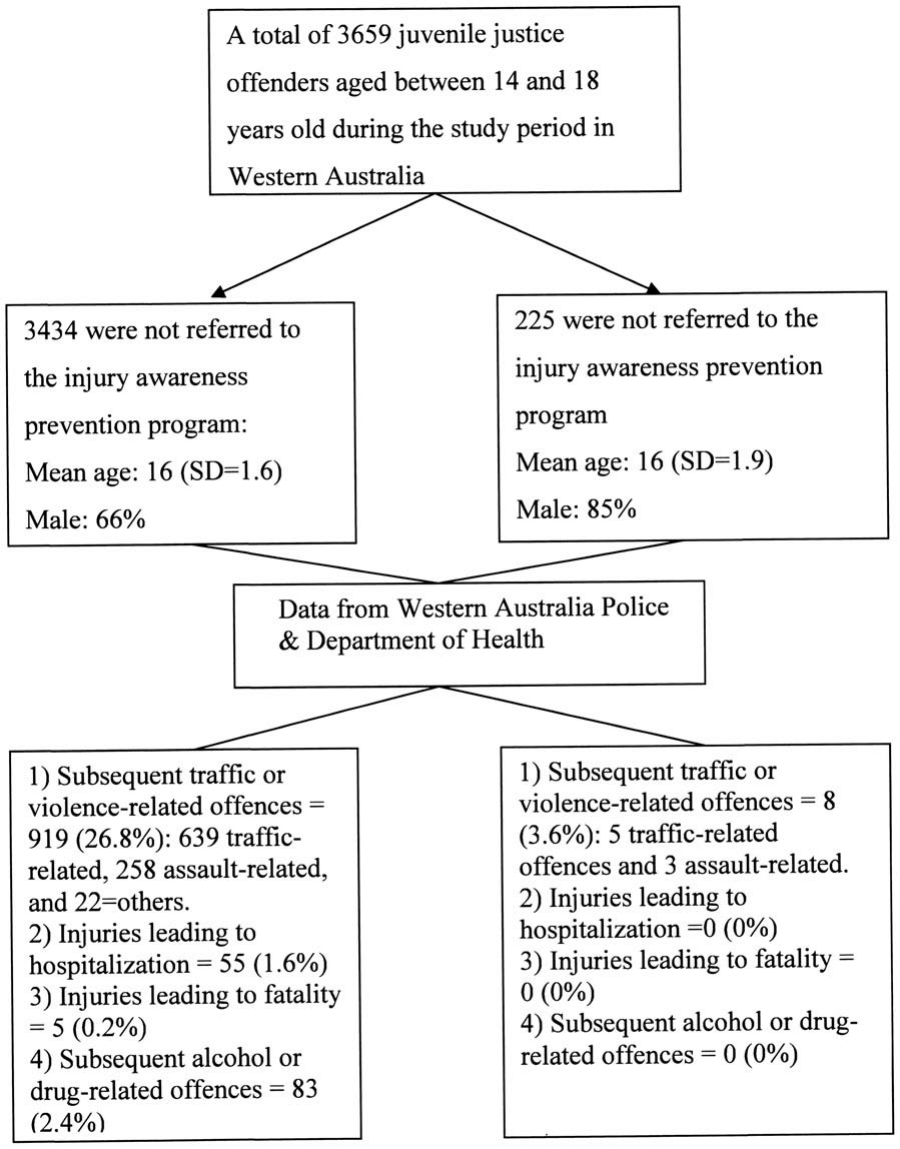
Flow chart showing data sources and outcomes of the study subjects. SD, standard deviation.

The median follow-up period for the whole cohort was 33 months (IQR: 17–44 months). The incidence of subsequent traffic or violence-related offences was significantly lower for those who had attended the program compared to those who had not (3.6% *vs.* 26.8%; absolute risk reduction [ARR] 23.2%, 95% confidence interval [CI] 19.9%–25.8%; number needed to benefit = 4.3, 95%CI 3.9–5.1; p = 0.001), as were injuries leading to hospitalization (0% *vs.* 1.6% including 0.2% fatality; ARR = 1.6%, 95%CI 1.2%–2.1%) and alcohol or drug-related offences (0% *vs.* 2.4%; ARR 2.4%, 95%CI 1.9%–2.9%) (**Figure 1**).

**Figure 2:**
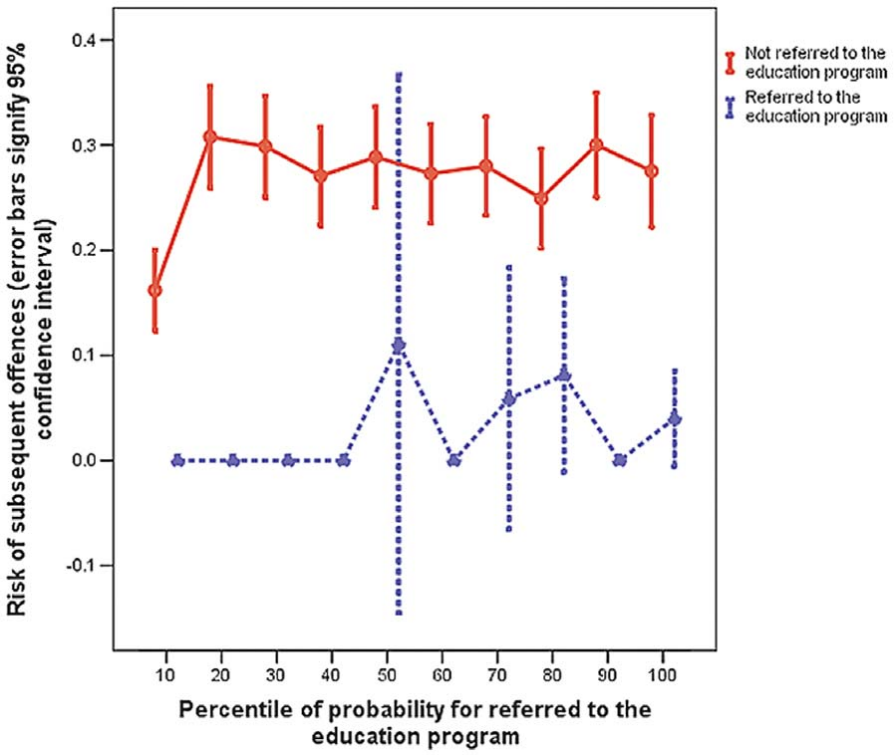
Interactions between effect of the education program on risk of subsequent offences and probability of being referred to the education program.

In the multivariate analysis, only P.A.R.T.Y. education program attendance (odds ratio [OR] 0.10, 95%CI 0.05–0.21) and a higher socioeconomic background (OR 0.97 per decile increment in IRSD, 95%CI 0.93–0.99) were associated with a lower risk of subsequent traffic- or violence-related offences (**Table 2**). The strength and direction of the association between attending the program and risk of committing subsequent offences was not changed by including the probability of being selected for attending the education program in the model (**Table 2**). The probability of selected or referred to the education program did not appear to have any relationship to the effectiveness of the program in reducing subsequent offences (**Figure 2**).

**Table 2:** Multivariate analysis showing the effect of attending the injury awareness program on risk of subsequent risk of committing traffic- or violence-related offences, with or without adjusting for potential selection bias for attending the program.

Variables	Odds ratio (95% confidence interval)	P value
*Model 1: without adjusting for potential selection bias*		
- Attendance of the education program	0.10 (0.05–0.21)	0.001
- Socioeconomic background (per decile increment in IRSD)	0.97 (0.93–0.99)	0.045
*Model 2: with adjustment for potential selection bias*		
- Attendance of the education program	0.10 (0.05–0.20)	0.001
- Socioeconomic background (per decile increment in IRSD)	0.97 (0.93–1.01)	0.055
- Number of offences prior to the study period	1.10 (0.96–1.25)	0.173
- Probability of selected to attend the education program (per 10% increment)	1.05 (0.90–1.22)	0.534

IRSD, Index of Relative Socioeconomic Advantage and Disadvantage. Age, gender, and ethnicity were removed during the modeling process because the associated p values were >0.25. The Hosmer-Lemeshow chi-square statistics of Model 1 and 2 were 8.0 (p = 0.334) and 9.1 (p = 0.337), respectively.

### Sensitivity analyses

The results remained unchanged after excluding individuals who had offences prior to the commencement of the study. P.A.R.T.Y. program attendance (odds ratio [OR] 0.10, 95%CI 0.05–0.19) and a higher socioeconomic background (OR 0.95 per decile increment in IRSD, 95%CI 0.92–0.99) were the only factors significantly associated with a lower risk of subsequent traffic- or violence-related offences. Similarly, the results also remained unchanged when including only Aboriginal subjects (OR 0.22, 95%CI 0.10–0.71, p = 0.012) or those from lower socioeconomic background (IRSD<median of IRSD of the whole cohort)(OR 0.11, 95%CI 0.04–0.31, p = 0.001).

## Discussion

This study showed that attendance of the P.A.R.T.Y. youth injury awareness program was associated with a change in the attitudes of the juvenile justice offenders about risk-taking behavior and significantly reduced their subsequent risk of injuries and committing traffic- or violence-related offences.

Trauma among young people remains as a major public health problem in many developed countries despite public health education and promotion [1]–[10]. Alcohol or drug abuse and risk-taking behaviors among young people have, in fact, become a dominant cause of trauma in many developed countries, including Australia [8], [9]. Reducing risk-taking behaviors of young people is difficult, and a multi-modality approach has been suggested to be the most effective way in reducing alcohol or drug-impaired driving and its consequences [10]. This study has established that this one-day injury awareness prevention program was effective in reducing risk-taking behaviors and injuries among juvenile justice offenders and requires careful consideration. First, the written feedback of participants suggests that an education program involving real-life scenarios and direct contact with trauma patients is an important component of this injury awareness education program. The ability of the participants to put themselves into the situation of a trauma patient and experience the potential implications of disability for them and their families had a strong and sustained effect on their attitude on risk-taking behaviors.

Second, our study showed that a program designed to change attitudes to risk-taking behaviors (57% vs. 21%) can successfully empower young people, reducing their subsequent risk of committing violence- or traffic-related offences, injuries and death. Third, this education program is relatively simple and inexpensive and should be able to be implemented widely, potentially changing the lives of many young people and their families. Furthermore, the average costs of each traffic-related fatality, serious injury, and minor injury in 2003 were US$1.8 million, $400,000 and $15,000, respectively, in Western Australia [6]. The estimated running cost of this education program was about US$38,000 per year. Assuming a discount rate of 3% per year, the cost per life-year gained of running this education program was estimated to be $14,628, making this education very cost-effective from an economic perspective.

The final consideration is the limitations of the study. First, this study involved only a cohort of juvenile justice offenders and whether this education program is equally effective for young people who have not committed an offence remains uncertain. The results of the Canadian study on high school students are very encouraging [12] and a prospective study on the effectiveness of this education program on a large cohort of high-school students is underway in this center. Second, although we have adjusted for potential selection bias by using a propensity score in the multivariate analysis, residual confounding is still possible from hidden or unmeasured biases related to the attitudes of the magistrates or young offenders. Third, we have used the Western Australia Police and Department of Health of Western Australia databases to capture subsequent offences and injuries or deaths of all juvenile justice offenders and, as such, we cannot exclude a small proportions of the study subjects would have left Western Australia to other states of Australia. However, the overall annual emigration rate of Western Australians is relatively low (<2.8%) [18], making this factor unlikely to compromise the validity of our results. Finally, the follow-up period of this study was relatively short (median 33 months). The long-term benefits of this education program remain uncertain and will be examined in our future studies.

In summary, this study showed that participation in the P.A.R.T.Y. youth injury awareness program was associated with a very significant reduction in subsequent risk of committing violence- or traffic-related offences, injuries, and death for juvenille justice offenders. However, due to the design of the study, hidden or unmeasured selection biases might have, in part, contributed to the dramatic beneficial effect of this education program on the study participants. Our results strongly support an increased implementation of this education program for junvenile justice offenders as part of their rehabiliation program.

## Supporting Information

File S1 Some of the written comments of the participants of the education program.
(DOC)
