# Supplementary material for: Effect of an Injury Awareness Education Program on Risk-Taking Behaviors and Injuries in Juvenile Justice Offenders: A Retrospective Cohort Study
Source: PLoS One. 2012 Feb 15;7(2):e31776. doi: 10.1371/journal.pone.0031776 (PMC3280207; doi:10.1371/journal.pone.0031776)
Supplement: File S1 — Some of the written comments of the participants of the education program. (DOC) [file pone.0031776.s001.doc]

Supporting Informtion file

Some of the written comments of the participants of the education program:

“I am thankful for the opportunity to attend it because it really opened my eyes to the consequences and repercussions of my actions. Also to be just as wary of others around myself because the decisions they make will affect you just as your decisions will affect others.”

“The party program really does, in my opinion, make you think twice about your decisions and the consequences. It makes you take that split second to make the right decision and not the risky decision. I feel as if this party program really made me think and change my way of thinking when it comes to risky decision making.”

“It seemed to really sink into the heads of most of the people in the program, which to me shows that the course is very effective. Being my own victim of my accident, since then I drive abiding to the laws, and don’t take any risks in which could leave me facing some dire consequences.”

“I would like to start of with saying the party program was very insightful, graphic and had a sense of extreme realty. This program is quite effective and I actually think it should be compulsory for high school students to attend.”

“The P.A.R.T.Y Program was an experience like no other. I thought it was excellent and extremely eye opening and life changing.  As I left the hospital I thought to myself I will never do anything to risk to myself or others whilst on the road. Lastly I would like to say that if everybody getting their driver’s license was made to attend the program then I feel that the number of young fatalities on the road would drastically drop.”

“Attending this program has made me realize how lucky the passengers in my car and myself were to not be injured and how our lives could have changed very badly because of the accident. I would not want to be responsible for the death or an injury to someone. Even though some of what I saw was frightening and graphic, it was a real wake-up call to me to be a responsible driver and to think more sensibly about my actions not only on the road but in life in general.”

“It doesn’t mean I won’t be going out and having a good time just I’ll be doing things that are more sensible compared to what I thought might have been OK, I know now that there’s no point taking the risk just because someone tells me that it’ll be OK.”

“I am just letting you know how valuable I think this was to me. I reckon every young driver should attend this course as it did show me and make me think of my choices I make while driving a car and the consequences it does have to lots of people.”

“After the program ended it has made me more cautious on the road, more aware of everything and the surroundings around me because I now know how serious things can get when you cross the stupid line. Thank you for sending me to this program it has made me aware of how quick things can go from good to bad when you let your concentration lapse. I do not want to become a statistic and I will abide by all road rules so nothing bad happens to me or anyone else.”

“Before today I didn’t even think about what could happen if I had a car accident or what damage I could cause to another person from doing something stupid. When I was looking at the man who fell off a motorbike, just laying there in the bed, I was thinking that I didn’t want to be him and that I didn’t want to put another person in that situation. I think today has really helped me in many ways.”

“I think the PARTY Program was good because it was interactive and real. Talks from real victims have more impact than just being talked to by professionals. It was quite brave of them to take part. From the PARTY Program I learnt that my actions on the road could have terrible consequences and affect my family, myself and the community.”

“This program should be compulsory just like sex education in school. I truly believe it can make a difference. I only wish my boyfriend went to this program as well. I tell him off all the time now and don’t reply to him when he texts message to me while driving.”

“I think I can take away a lot from the program and apply it to my daily life, changing my attitude towards driving and even having a greater respect for people who are paraplegic and the process which they have to go through, the recovery alone being an epic battle. The PARTY program has altered my attitude towards driving and my attention to the road.”

“Thank you very much for allowing me to come to the PARTY program seminar, it has changed me in so many ways, you cannot believe how cautious I will be on the roads, and am constantly telling my friends about the day I had at the hospital and what I learnt and saw.  That day has really changed me and I wanted to say thank you because if I hadn’t gone to the seminar I would still abuse my road privileges by not concentrating or speeding. I think a lot more young teenagers who are taking their drivers license assessment would value something like this to make them fully aware of what could happen to them or others on the road if they make a stupid decision.”

“I must say when I first arrived at Royal Perth hospital that morning the only thing I had going through my head was that this isn’t going to be any fun and very boring and uneventful. However, as the day went on, I learnt a lot and my eyes slowly started to open then it hit me that we should all think before we act and that we should all think of how it will affect others we know. The time in which we spent in the wards with the patients it was good but what I would find most fun and eye opening was to see the other side of the story - the effects of an accident on the loved ones of the patients because this let me know just how bad things can affect people around me.”

 
